# Acute effects of therapeutic 1-MHz ultrasound on nasal unblocking of subjects with chronic rhinosinusitis

**DOI:** 10.1590/S1808-86942011000100002

**Published:** 2015-10-19

**Authors:** Wanize Almeida Rocha, Kátia Maria Gianezeli Rodrigues, Rowdley Robert Rossi Pereira, Breno Valentim Nogueira, Washington Luiz Silva Gonçalves

**Affiliations:** 1 Espirito Santo Federal University (Universidade Federal do Espírito Santo - UFES). Coordinator of the physical therapy course at the Novo Milenio School (Faculdade Novo Milênio - FMN); 2 Novo Milenio School (Faculdade Novo Milênio - FMN); 3 Master's degree in orthodontics. Rio de Janeiro Federal University (Universidade Federal do Rio de Janeiro - UFRJ). Collaborating professor at the Dental Clinic Department (Departamento de Clínica Odontológica), UFES; 4 Adjunct professor of the Morphology Department, UFES; 5 Collaborating professor of the Physiology Department, UFES

**Keywords:** physical therapy (specialty), rhinitis, sinusitis, ultrasonic therapy

## Abstract

Low-intensity ultrasound therapy (LIUST) has been described as a plausible treatment for chronic rhinosinusitis (CRS).

**Aims:** To evaluate the short-term effects of continuous 1MHz LIUST on nasal obstruction in subjects with CRS.

**Material and Method:** A cohort placebo-controlled study comprising 26 CRS adults (10 men, 16 women), sequentially allocated into two groups: control-placebo (CP, n= 12) and treated with LIUST (US, n= 14). The treatment consisted of: ISATA = continuous 1MHz, 1W.cm-2 for four minutes in the maxillary sinuses and nasal septum. The equipment was switched off in the CP group. The degree of obstruction was assessed by the total volume of secretion expelled (VSEx) after nasal instillation of 5 mL saline solution (NaCl-0.9%) followed by nasal lavage. The volume of expired air (VEA) was assessed with a Glatzel mirror.

**Results:** The data showed an increase (p<0.01) in VSEx and VEA after ultrasound therapy, suggesting a 64% improvement of nasal obstruction compared with the CP group.

**Conclusions:** Continuous LIUST reduced nasal obstruction and congestionç it may be used effectively in the respiratory therapy of CRS patients.

## INTRODUCTION

Rhinosinusitis may be described as inflammation of the nasosinusal mucosa due to infection, trauma, exposure to chemicals or allergens.^1,^^2^ Sinusitis without rhinitis is rare, but rhinitis may occur alone. Non-specific chronic infectious rhinitis is often associated with sinusitis, and should be classified and treated as rhinosinusitis.^3,^^4^ Voegels et al.^5^ reported that the prevalence of nasosinusal infection is high, and that it requires complex and prolonged therapy, particularly in the first and second decades of life.

The pathophysiology of rhinosinusitis consists of obstruction of paranasal sinus ostia (which aerate and drain the sinuses).^5,^^6^ Occlusion leads to intrasinus hypoxia and hypercapnia, vasodilation, increased capillary permeability, interstitial edema, hypertrophied mucosae, transudation of fluids and decreased mucociliary function. Microorganisms grow in this environment within the sinus, which increases inflammatory reactions.^6,^^7^ Anatomical variations of the nasal septum, middle turbinates and uncinate process are predisposing factors because they result in stenosis and may impede ventilation, which worsens the condition.^1,^^7^

Rhinosinusitis is usually time-defined according to frequency and duration of disease;^1^, ^2^, ^3^, ^4^ it is commonly classified as four subtypes: acute (sudden onset, lasting up to four weeks), recurring (over four yearly episodes with full resolution between each), subacute (persisting after four weeks and remaining for over 12 weeks, but less intensely), and chronic (persisting for over 12 weeks).^1,^^8^ Signs and symptoms are similar in any subtype; the clinical picture is what varies. There is rhinorrhea, significant nasal block, dry coughing that worsens at night, throat clearing, cacosmia, halitosis, hyposmia, and nasal bleeding in the chronic phase.^1^, ^2^, ^3^, ^4^, ^5^, ^6^, ^7^, ^8^

Predisposing factors - such as duration and frequency of crises - are relevant for defining appropriate therapy in chronic rhinosinusitis.^1^, ^2^, ^3^, ^4^, ^5^, ^6^, ^7^, ^8^ Recent studies have recommended using systemic or topical drugs and adjuvant therapy.^1,^^3,^^8^ Aspiration of nasal sinuses is indicated if hosts are immunocompromised, severely ill, or if antibiotic therapy is ineffective.^8^ Other studies have proposed using low intensity ultrasound therapy (LIUST) applied on the nose (maxillary and frontal sinuses).^9^, ^10^, ^11^, ^12^, ^13^

Ultrasound (1 to 3-MHz) is low power and low intensity mechanical energy which is produced by passing a high frequency electrical current through a piezoelectric material.^14^, ^15^, ^16^ Sound waves propagate longitudinally in biological tissues; particles vibrate parallel to the direction of waves.^15,^^16^ The literature describes this continuous ultrasound wave as being able to increase the temperature of tissues - thereby causing thermal effects. Pulsed ultrasound waves act on a cell and/or molecular level by altering membrane permeability, ionic concentration gradients and cell biochemical activity.^14^, ^15^, ^16^, ^17^ More recently, ultrasound has been employed as adjuvant therapy in several conditions such as tissue repair,^16^ reduction of muscle and joint pain,^17,^^18^ modulation of inflammation,^19,^^20^, ^21^, ^22^ and reduction of tendon adhesions.^17^, ^18^, ^19^ A few studies have demonstrated that LIUST-induced accelerated skin tissue repair results from local modulation of cell mediators/signalers such as nitric oxide, histamine and other cytokines that are involved in inflammation in healing; it also increases the number of fibroblasts and collagen synthesis.^16^, ^17^, ^18^, ^19^ There are few published clinical studies on LIUST as treatment or adjuvant therapy for chronic rhinosinusitis;^9^, ^10^, ^11^, ^12^ it seems plausible that LIUST may reduce nasal block and congestion by its physical and chemical effects on tissues,^13,^^21,^^22^ reestablishing interstitial mucosal permeability, reducing the viscosity of paranasal sinus secretions and helping eliminate nasal discharges.^13^, ^14^, ^15^ Thus, the main purpose of this study was to evaluate the short-term effects of continuous 1 MHz LIUST on nasal block and the symptoms of subjects with chronic rhinosinusitis.

## METHOD

An experimental study was carried out according to Brazilian and international standards for research in human beings (Regulation 196/96-CNS/OMS and others); the institutional review board approved this study (no. 021/2007).

### Study Design

A placebo-controlled experimental pre- and post-test study was carried out to evaluate the short-term effects on nasal obstruction of continuous 1 MHz LIUST in subjects with chronic rhinosinusitis. The sample comprised 26 patients with chronic rhinosinusitis that had undergone medical treatment for at least 12 weeks in an ENT outpatient unit at our institution, and that were referred to the physical therapy unit, from 1 July 2007 to 28 September 2008.

### Enrollment/referral

The inclusion criteria were as follows: patients of both sexes, aged from 18 to 60 years, diagnosed with rhinosinusitis according to the Brazilian guidelines for this condition, treated medically for at least 12 weeks, currently not using systemic or topical (nasal) medication for at least one week, and upon signing a standard free informed consent form confirming their voluntary participation.

Exclusion criteria were: presence of chronic diseases (diabetes, arterial hypertension), smoking, fever, skin diseases of the face (such as acne), nasal polyps, tumors or cysts (as verified with computed tomography), other facial skin lesions or allergies, and diseases that contraindicated LIUST or that might affect the final results. Participants were asked to maintain their usual diets, to refrain from drinking caffeine-containing and alcoholic beverages, and to avoid taking systemic and/or topical nasal medication (especially sympathomimetic drugs) in the preceding 24 hours before gathering data.

### Groups and study protocol

After meeting enrolment criteria, 26 patients diagnosed with chronic rhinosinusitis (10 male and 16 female) were referred to the physical therapy unit and allocated sequentially to two groups by arrival order: odd numbered patients comprised the placebo-control group (CP group, n= 12) and even numbered patients comprised the treated group - 1-MHz ultrasound (US group, n= 14). All patients underwent the same procedures, done by the same researcher; but the ultrasound probe was not connected to the ultrasound device (AVATAR IV^®^ - KLD - Biosystems - São Paulo, Brazil) during the procedure. An evaluation by a physical therapist was carried out to investigate possible contraindications to LIUST on the face (maxillary sinuses and nasal septum).

In a ventilated room at 24^o^C during the afternoon, the participant's vital signs and body weight and height (and body mass index) were measured. Subjects were asked to quantify their current main symptoms of chronic rhinitis (facial pain, nose block, rhinorrhea, throat clearing, coughing and epistaxis) on a 0-10 visual analog scale.

The study protocol was started, consisting of placing subject in dorsal decubitus (head aligned longitudinally with the body) during 20 minutes for facial asepsis with topical antiseptics. Next, the total volume of secretions expelled (VSEx, mL) was measured; this was measured by instilling simultaneously 2.5 mL of saline (NaCl - 0.9%) in each nostril, collecting the expelled nasal lavage and storing for quantification. The degree of nasal obstruction and/or permeability was measured by the volume of air expelled (VAEx, cm^2^) as measured using a Glatzel mirror.^23,^^24^

After measuring the VSEx and VAEx, ultrasound gel (H_2_O) was placed over the maxillary sinuses and nasal septum, and low-intensity ultrasound was applied according to the following parameters: I_SATA_ (spatial average temporal average intensity) = 1-MHz, 1.0 W.cm^-2^, continuous mode, for 4 minutes. The 1-MHz ultrasound probe was systematically moved in circles over the maxillary sinuses and the nasal septum during 4 minutes.^11,^^20^ After 20 minutes of LIUST over the nose, the VSEx and VAEx were again measured, and the symptoms of chronic rhinosinusitis were quantified again on a visual analog scale in both groups. The 1-MHz ultrasound probe insonates over an effective area of 0.5 cm^2^; this was checked and calibrated before the study on precision scales at the Engineering Department of the institution. The ultrasound insonation level (W.cm^-2^, 5%) in this study is the amount recommended for therapeutic ultrasound by the WFUMB (Word Federation Ultrasound Medicine and Biology) and the FDA (Food and Drug Administration).^14^

### Data analysis

The sample size was calculated using the Statemat^®^ 2.0 software, which indicated an n = 24 subjects (patients with chronic rhinosinusitis) as an ideal sample for 90% test power and 0,05 alpha error. The variables were considered normal in the Shapiro-Wilk test, and values were expressed as means ± standard deviation (SD). The paired and non-pared Student's t test was applied to check differences among variables. Two-way analysis of variance (ANOVA) was applied to establish differences between the two groups, followed by the Tukey multiple comparison test (post hoc). The minimum significance level for the differences was p ≤ 0,05. The Prism 5.0^®^ (GraphPad, San Diego, CA, USA) software was used for these analyses.

## RESULTS

Table 1 shows the general features, body composition, and vital signs of subjects with chronic rhinosinusitis; there were no significant differences among these parameters in the CP and US groups (1-Mhz therapeutic ultrasound), characterizing the sample as homogeneous.

**Table 1:** General features, body composition, and vital signs of subjets with chronic rhinosinusitis

Variables	CP (N= 12)	US (N= 14)	p-value
Male/Female	5 / 7	5 / 9	
Age (years)	26 ± 9	27 ± 13	0,212
Weight (Kg)	78 ± 2	72 ± 4	0,134
Body-mass index (Kg.cm-^2^)	30.3 ± 3	28.6 ± 4	0,097
Mean arterial blood pressure (mmHg)	102 ± 4	105 ± 1	0,163
Heart rate (bpm)	90 ± 3	92 ± 4	0,127
Respiratory rate (rpm)	18 ± 2	20 ± 3	0,118

Values are expressed as means ± standard deviation (SD). Student's t test.

Table 2 shows the main symptoms in chronic rhinosinusitis subjects before and after applying 1-MHz nasal LIUST (US group) or placebo (CP group). Except for coughing and epistaxis, chronic rhinosinusitis symptoms regressed significantly in the US group compared with the CP group.

**Table 2:** Main symptoms of chronic rhinosinusitis before and after a single use of 1-MHZ ultrasound (US) or placebo (CP) in both groups

Symptoms (visual analog scale or VAS)	CP (n = 12)		US (n = 14)	
	Pre	Post	Pre	Post
Facial pain	7,9 ± 0,2	7,3 ± 0,5	7,5 ± 0,5	2.1 ± 0,2**++
Nose block	8,3 ± 0,2	8,0 ± 0,3	7,9 ± 0,4	3,0 ± 0,5**++
Rhinorrhea	5,9 ± 0,3	6,3 ± 0,4	6,1 ± 0,3	2,0 ± 0,2**++
Throat-clearing	5,0 ± 0,4	4,0 ± 0,5	4,9 ± 0,3	2,7 ± 0,3*+
Coughing	1.8 ± 0,2	2,1 ± 0,3	1,9 ± 0,2	1,6 ± 0,4
Epistaxis	1,6 ± 0,1	1,8 ± 0,2	1,6 ± 0,2	1,4 ± 0,3

Values are expressed as means ± standard deviation. Visual analog scale or VAS (0-10). CP = placebo-control group, US = therapeutic ultrasound group, Student's t test for paired samples, two-way analysis of variance (ANOVA) followed by the Tukey multiple comparison test (post hoc).

*p ≤ 0,05 e **p ≤ 0,01 for Pre- vs. Post-;

+*p* ≤ 0,05 + +*p* ≤ 0,01 for CP vs. US.

**Figure 1:**
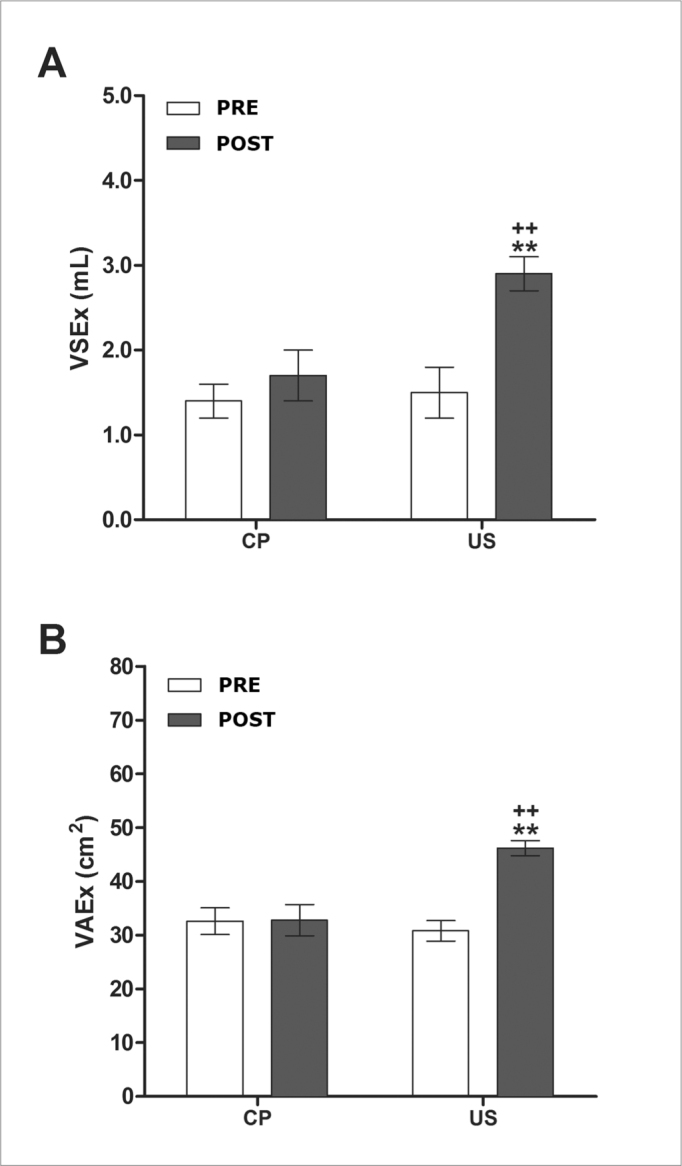
Effects of low-intensity ultrasound therapy (LIUST) on nasal block in subjects with chronic rhinosinusitis. Panel (A), volume of nasal secretion expelled (VSEx, mL). Panel (B), volume of air expelled (VAEx, cm^2^) in the placebo-control (CP) group, and 1-MHz LIUST (1W.cm-2) insonated (US) groups - 1-MHz LIUST (1W.cm-^2^). Values are presented as means ± standard deviation. Student's t test for paired samples, two-way analysis of variance (ANOVA), followed by the Tukey multiple comparison test (post hoc). *p ≤ 0.05 and **p ≤ 0.01 for Pre- vs. Post-; +*p* ≤ 0.05 + +*p* ≤ 0.01 for CP vs. US.

Figure 1A shows that VSEx values in the US group increased after LIUST (*p* ≤ 0,01), as opposed to these values in the CP group. Figure 1B shows a similar increase in VAEx values in the US group (*p* ≤ 0,01). This may be translated as a 64% decrease in nasal obstruction and congestion in chronic rhinosinusitis subjects treated with continuous 1-MHz LIUST, compared with subjects in the CP group. There were no significant pre- and post-test differences when applying LIUST with the probe switched off (placebo), as expected.

## DISCUSSION

This study presents the short-term effects of nasal continuous mode 1 MHz LIUST on nasal block and other symptoms of chronic rhinosinusitis. Our data showed that a single 4-minute application of 1 MHz LIUST at a 1.0 W.cm^-2^ (SATA) dose on maxillary sinuses and the nasal septum yielded a significant increase in VSEx and VAEx values; it also resulted in a significant regression of other symptoms of chronic rhinosinusitis in the study group.

Our findings are similar to those of Naghdi et al.^12^ and Ansari et al.^9,^^10^ who showed that the frequency of the main symptoms in chronic rhinosinusitis (facial pain, nasal congestion and nasal block, rhinorrhea, olfaction disorders, and coughing) decreased significantly following 1 and 3 MHz LIUST. These studies showed that the thickness of the sinus mucosa decreased, the meatal ostia became more open, and paranasal sinus lesions regressed after the 15^th^ day of LIUST at 1.0 W.cm^-2^ (SATA) for 5 minutes over the maxillary and frontal sinuses.^10,^^12^ Studies by Barros et al.^11^ demonstrated that only two sessions of pulsed mode LIUST at 1.0 - 1.5 W.cm^-2^ for 3 minutes were not enough to decrease nasal block in 13 subjects with rhinosinusitis - with or without antibiotics. However, this approach significantly reduced other symptoms such as headaches and facial pain in these subjects.

We found that continuous mode 1 MHz LIUST at 1.0 W.cm^-2^ for 4 minutes over the maxillary sinuses and nasal septum not only reduced nasal block and congestion by 64%, but also resulted in regression of the main symptoms of chronic rhinosinusitis (Table 1). It is worth pointing out that the short-term effects of nasal decongestion and relief of other symptoms in 90% of patients undergoing LIUST lasted during the ensuing two hours, which confirms the therapeutic potential of LIUST in chronic rhinosinusitis.

Improvements in VSEx and VAEx levels - regression in nasal block and congestion and other symptoms such as facial pain, rhinorrhea and throat-clearing - in patients with chronic rhinosinusitis following a single application of LIUST was probably due to the mechanical effects (vibration)^14^, ^15^, ^16^ of ultrasound on the nasal mucosa and discharge. Studies have shown that therapeutic ultrasound changes the thixotropy in the nasosinusal mucosa, which is the ability of a semisolid material (mucus) to progressively become fluid because of mechanical agitation and to return to its original state after a period of rest.^14^, ^15^, ^16^,^25^ Studies have shown that the nasosinusal mucus is partially thixotropic; it does not return to its original rheologic state after mixing has ceased.^25^ This correlates with our findings, as VSEx values increased significantly, which suggests that there was a mucolytic effect that resulted in elimination of nasal secretions by lavage, as observed in patients with chronic rhinosinusitis.^25^, ^26^, ^27^, ^28^

Similarly, the observed effects on VSEx and VAEx and on other symptoms of chronic rhinosinusitis may have taken place because of an increased local temperature - the known thermal effects of continuous LIUST. These effects also change the viscoelastic (rheologic) properties of the nasosinusal mucus by breaking the disulphide bonds of mucoproteins^26^, ^27^, ^28^ thereby changing the fluidity of nasal secretions, which therefore drain more easily after nasal lavage in the LIUST-treated group.^13^, ^14^, ^15^,^25^, ^26^, ^27^, ^28^ Nasal lavage with saline solution only - without LIUST - did not have the same effect on VSEx, VAEx, and on other symptoms of chronic rhinosinusitis in the placebo-control.

The direct effects of low-intensity ultrasound on biological tissues cannot be discarded;^14,^^16^ these effects may affect cell membrane permeability,^15^, ^16^, [Bibr bib17][Bibr bib20][Bibr bib21] enzyme activity and inflammation,^19,^^21,^^29^ and may facilitate local fibrinolysis.^17^, ^18^, ^19^, ^20^, ^21^, ^22^ LIUST has been shown to modify cellular and humoral signaling factors, especially the bioavailability of nitrous oxide, thereby regulating interstitial permeability and tissue fluid balance;^9,^^12,^^13,^^29^ this in turn results in decongestion of the nasopharyngeal mucosa, regression of interstitial edema and of nasal obstruction/congestion in patients with chronic rhinosinusitis. Supporting this assumption, recent reports in the literature have shown strong correlations among nasal mucosa congestion rates, fibrosis, and vascular density in patients with chronic rhinosinusitis.^30^ Thus, fibrosis and vascular density are directly associated with the level of congestion of the nasal mucosa.

## CONCLUSION

Based on our results and the literature, we conclude that applying continuous 1-MHz LIUST at 1 W/cm^2^ during 4 minutes over the maxillary sinuses and nasal septum results in decreased nasal obstruction and improved nasal airflow. The clinical outcome in patients with chronic rhinosinusitis is improved and new possibilities open up for LIUST in respiratory physical therapy. Notwithstanding these clinical benefits, we underline the need for additional studies using different parameters to characterize these therapeutic mechanisms in more detail, and to find possible adverse effects of therapeutic LIUST in chronic rhinosinusitis. Furthermore, patients should be carefully assessed to avoid wrong diagnoses and delayed treatment, thereby avoiding complications of the disease.

## ACKNOWLEDGEMENTS

The authors wish to thank the participants and the healthcare professionals in the ENT unit of the Casssiano Antônio de Morais University Hospital – HUCAM/ UFES for their valuable collaboration; also the electrical engineer Manoel Ramos Penha for help in measuring, calibrating and maintenance of the ultrasound device that was used in this study.
